# Fast-Acting Sublingual Zolpidem for Middle-of-the-Night Wakefulness

**DOI:** 10.1155/2014/527109

**Published:** 2014-02-05

**Authors:** Joseph V. Pergolizzi, Robert Taylor, Robert B. Raffa, Srinivas Nalamachu, Maninder Chopra

**Affiliations:** ^1^Department of Medicine, School of Medicine, Johns Hopkins University, Baltimore, MD 21205, USA; ^2^Department of Anesthesiology, Georgetown University School of Medicine, Washington, DC 20057, USA; ^3^Department of Pharmacology, School of Medicine in Philadelphia, Temple University, PA 19140, USA; ^4^NEMA Research, Inc., Naples, FL 34108, USA; ^5^School of Pharmacy, Temple University, Philadelphia, PA 19140, USA; ^6^Kansas University Medical Center, Kansas City, KS 66160, USA; ^7^International Clinical Research, Leawood, KS 66211, USA; ^8^Kirax Pharmaceuticals, Bonita Springs, FL 34134, USA

## Abstract

Sleep disorders (somnipathies) are conditions characterized by disruptions of sleep quality or of sleep pattern. They can involve difficulty falling asleep (prolonged sleep onset latency), difficulty staying asleep (disturbance of sleep maintenance), sleep of poor quality (unrefreshing), or combinations of these and can lead to poor health and quality of life problems. A subtype of sleep-maintenance insomnia is middle-of-the-night wakefulness, a relatively common occurrence. Zolpidem, a nonbenzodiazepine benzodiazepine receptor agonist, allosterically modulates an ion channel and increases the influx of Cl^−^, thereby dampening the effect of excitatory (sleep disrupting) input. Recently, product label changes to some zolpidem containing products have been implemented by the FDA in order to reduce the risk associated with their morning after residual side effects. A new formulation of zolpidem tartrate (Intermezzo) sublingual tablet, an approved product indicated exclusively for the treatment of middle-of-the-night wakefulness and difficulty returning to sleep, did not have its label changed. We present a short summary of its basic science and clinical attributes in light of the recent regulatory changes for zolpidem products.

## 1. Introduction

**Figure 1:**
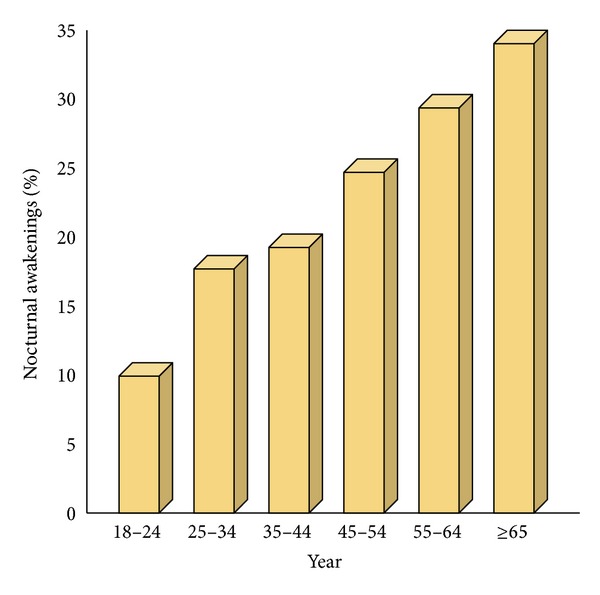
The prevalence of nightly nocturnal awakenings by age group (based on data from Ohayon) [64].

Middle-of-the-night wakefulness (sleep-maintenance insomnia), a core symptom of insomnia, is a somnipathy that affects about 30.0% of those with incident chronic insomnia at baseline and 18.0% of the general population [1] and may contribute to another core symptom of insomnia, unrefreshed sleep [2]. See Figure 1. Certain populations may be more severely impacted: 78.3% of chronic pain patients have difficulty staying asleep [3] and women between the ages of 40 and 59 years rate “awakening during the night” as their most frequent symptom of insomnia [4]. Although there are many pharmacological and nonpharmacological options to treat sleep disorders, there had previously been no sleep aid indicated specifically for middle-of-the-night wakefulness. In fact, previous products were unsuited for this use because they are long acting and, if taken in the middle of the night, would require the patient to sleep for several hours past their normal wake time. It is known that relatively few insomniacs seek medical care [5] and those that do may still have symptoms a year later [6]. The reasons for this are unclear but may owe, at least in part, to the fact that patients suffering from middle-of-the-night wakefulness had no pharmacological options. This narrative review discusses a new formulation of the established pharmacological sleep aid zolpidem (Intermezzo, Transcept/Purdue Pharma cleared for market by the Food and Drug Administration in November 2012), the first sleep aid indicated exclusively for the treatment of middle-of-the-night wakefulness and difficulty returning to sleep.

## 2. Sleep and GABAergic Transmission

**Figure 2:**
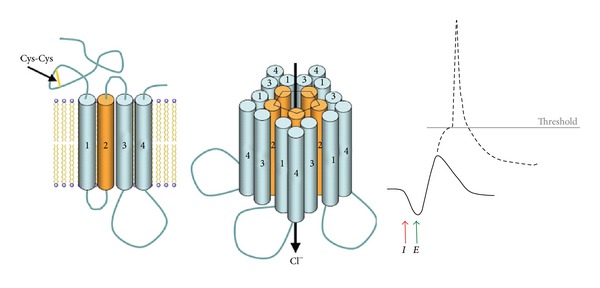
The pentameric structure of the GABA_A_ receptor is composed of 4-transmembrane monomeric subunits (a disulfide bond in the *N*-terminal extracellular domain, characteristic of the family of cys-loop receptors which includes the GABA_A_ receptor, is depicted). Zolpidem allosterically enhances Cl^−^ influx, which hyperpolarizes the neuronal transmembrane potential (at I), thereby making it less likely to fire in response to excitatory input (at E).

In coordination with other neurotransmitters and neuromodulators, the inhibitory amino acid GABA (*γ*-aminobutyric acid), acting through the GABA_A_ receptor, plays an important role in sleep/wake cycles [7–9]. The GABA_A_ receptor is a ligand-gated ionotropic type receptor. Binding of the endogenous neurotransmitter GABA or an exogenous agonist analog increases CI^−^ ion influx down its concentration difference from the extracellular side to the intracellular side of neurons. Since the transmembrane resting potential is already negative, the influx of CI^−^ hyperpolarizes the neuron, that is, increases the transmembrane potential difference, producing a postsynaptic inhibitory potential (IPSP). The hyperpolarization means that the neuron is less likely to fire in response to an excitatory input. See Figure 2.

## 3. Benzodiazepines

Benzodiazepines have been the go to choice for short term treatment of insomnia. Benzodiazepines work by binding to the GABA receptors causing inhibition of neuronal excitation and result in increased total sleep time and shortened sleep latency [10, 11]. There are a number of FDA approved benzodiazepines for insomnia and they are generally considered safe and effective when used at recommended doses and for short term. However, their mechanism of action and long half life have been linked with a number of adverse effects. Common reported adverse effects include changes in sleep architecture, daytime hangover, psychomotor impairment, rebound insomnia, withdrawal, drowsiness, dizziness, and headache [10, 11]. Daytime hangover has received much attention due to the increased association with impairing some daytime activities such as driving, resulting in many motor vehicle accidents [12]. To provide a safer treatment option for patients, recently nonbenzodiazpines (also known as z-hypnotics) have entered the market.

## 4. Zolpidem Pharmacodynamics 

Chemically, zolpidem is an imidazopyridine not a benzodiazepine, but zolpidem acts as a benzodiazepine receptor agonist. It has a high binding affinity for the benzodiazepine receptor, which acts as a positive allosteric modulator of the GABA_A_ receptor. Benzodiazepine receptor agonists, such as zolpidem, increase the neuronal transmembrane influx of Cl^−^ ions [13], which results in neuronal hyperpolarization and decreased neuronal excitability [14].

A large number of studies indicate that zolpidem has selective binding affinity and functional efficacy at the GABAA receptors containing *α*1 subunits [15]. Genetic studies of knock-in mice suggest the main target of zolpidem, GABAA receptors with *α*1 subunits, are involved in sedative action, while GABAA receptors with other subunits are associated with a variety of other activities, such as anxiety, myorelaxation, and cognitive function [16]. Similar genetic studies suggest that sleep continuity, as opposed to the motor sedative action, is mediated by subunits other than *α*1 [17]. Thus, the molecular mechanism underlying the clinical efficacy of zolpidem's hypnotic action is likely to be more complex, involving both *α*1 and other subunits.

Zolpidem is the active ingredient in several products commercially marketed to treat insomnia (see Table 1).

## 5. Pharmacokinetics of Sublingual Formulations

Two formulations of sublingual tablets are available: a higher-dose product (Edluar, 5 and 10 mg) indicated for difficulty with sleep onset and a fast-acting low-dose product (Intermezzo, 1.75 and 3.5 mg) indicated for middle-of-the-night wakefulness [18]. The low-dose product is available as a lozenge to be placed under the tongue and allowed to dissolve. A bicarbonate-carbonate buffer speeds absorption through the oral mucosa. Patients should be advised not to chew the lozenge or swallow it whole [18, 19]. Another formulation as an oral spray is available, which is considered bioequivalent to the fast-acting sublingual tablets [20]. Key pharmacokinetic properties of other zolpidem products appear in Table 2.

Doses of 1.75 and 3.5 mg produce a plasma concentration of >20 ng/mL within about 20 minutes, the level associated with sedation, and maintain it for about four hours [18]. Maximum serum concentration (*C*_max⁡_) is achieved in about 35–75 minutes with a half life of 2.5 hours. In normal healthy subjects (ages 21 to 45) the average  *C*_max⁡_  and AUC values for 3.5 mg Intermezzo were 77 ng/mL and 296 ng·h/mL, respectively, for women and 53 ng/mL and 198 ng·h/mL, respectively, for men (*C*_max⁡_  and AUC for 1.75 mg in women were 37 ng/mL and 151 ng·h/mL, resp.) [21]. At 12 hours, the drug is no longer detectable in plasma [22]. Thus, fast-acting low-dose zolpidem is associated with a rapid onset of short-term (four hours) sedation. This formulation does not appear to accumulate in the plasma, even after long-term administration [23].

The pharmacokinetic parameters, especially bioavailability, of sublingual zolpidem are variable depending on patient characteristics such as gender, age, or comorbidities. The pharmacokinetics of zolpidem differs by gender, resulting in gender-specific dosing [18, 24]. Gender differences in the clearance of cytochrome-P (CYP) 3A4 substrates have been put forth as a possible explanation for different drug metabolism by gender of zolpidem and other agents [25]. A study of genetic polymorphisms of CYP3A4 and CYP2C19 among the Chinese Han people found that zolpidem is poorly metabolized by some individuals in this group [26], indicating that genetic factors may also play a role in zolpidem metabolism. In elderly patients taking 3.5 mg of sublingual zolpidem,  *C*_max⁡_  and AUC_0−4 hr_ were higher by 34% and 30%, respectively, than in nonelderly subjects. The  *C*_max⁡_  and AUC in elderly subjects were lower compared to elderly taking 3.5 mg, but higher than nonelderly taking 1.75 mg [21]. In addition, patients who had hepatic impairment had mean  *C*_max⁡_  and AUC values two times and five times higher than patients with normal hepatic function when taking 20 mg oral zolpidem tartrate [21].

## 6. Efficacy

In a randomized, double-blind, placebo-controlled study, subjects with insomnia characterized by middle-of-the-night wakefulness were randomized into three groups [27]. Each group underwent a two-day treatment period followed by a five-to-12-day washout period. During treatment, subjects were awakened four hours after lights were turned off and administered one of the following: fast-acting zolpidem sublingual 3.5 mg or 1.75 mg or similar-looking placebo. Subjects were kept awake for 30 minutes and then allowed back to bed for another four hours. A total of 82 adults were enrolled and evaluated by both polysomnography and questionnaires about their sleep quality. All subjects crossed over into all three groups. Fast-acting low-dose sublingual zolpidem was found to confer a dose-related decrease in sleep latency to persistent sleep and total sleep time (*P* < 0.001) versus placebo and subjects taking the active agents did not exhibit impairment or sleepiness the following day. Fast-acting zolpidem sublingual 3.5 mg improved sleep quality (*P* < 0.001), ability to function, and level of refreshed sleep (both *P* < 0.05) versus placebo and zolpidem 1.75 mg also provided significantly higher levels of refreshed sleep versus placebo (*P* < 0.05).

## 7. Safety

In a randomized, three-arm, double-blind, placebo-controlled study conducted by Roth et al., adverse events were mild to moderate and reported by four, three, and seven subjects in the zolpidem 3.5 mg, zolpidem 1.75 mg, and placebo groups, respectively [27]. Events that were reported for active treatments included GI disorders, infections, urinary tract infection, glossodynia, increased blood pressure, nervous system disorders, headache, skin disorders, and contact dermatitis. Adverse events were not significantly greater in the active treatment arms.

In a double-blind, placebo-controlled study of 24 healthy volunteers, subjects were exposed to doses of 1.0, 1.75, and 3.5 mg of fast-acting sublingual zolpidem [22]. Subjects were randomized into each of the doses, separated by a washout period of 5–12 days. Medication was administered every morning at 8 AM and adverse events were assessed through length of study. A total of 48 adverse events (AEs) were reported, most of which occurred at higher doses. The most frequently reported AE was somnolence, reported by 12.5% of placebo subjects and 41.7% of subjects taking zolpidem 3.5 mg; fatigue was also reported at all doses. Dizziness and headache were reported by 4.2%, 12.5%, and 8.3% of the 1.75 mg, 3.5 mg, and placebo groups, respectively. Nausea was reported only at 3.5 mg doses (12.5%). One severe AE occurred and was deemed unrelated to treatment (epigastric pain; subject was in the 1.75 mg group). Two further AEs were considered unrelated to treatment (headache in the 1.75 mg group and dysmenorrheal in placebo) and were treated with acetaminophen and ibuprofen. All other AEs resolved without the need for treatment. Time of occurrence for AEs was not reported and statistical difference between placebo and active treatments was not conducted.

## 8. Morning Effects

Sleepdriving and other complex behaviors have been reported in the literature and popular media for other formulations of zolpidem [28–30], including reports of sleepdriving by prominent individuals [31]. Sleep-related complex behaviors associated with zolpidem have included sleep cooking, sleep eating, sleep shopping, and sleep conversations, usually associated with anterograde amnesia for the episode [32]. It is unclear why such behaviors occur. Enhancing GABA activity at the GABA_A_ receptors might trigger hypnosedative behaviors and amnesia, the latter possibly the result of consolidating short-term to long-term memory, but this explanation is inadequate. Dosing may play a role; in the 15 cases involving zolpidem in a study of complex sleep-related behaviors, 10 involved taking ≥10 mg at bedtime; however, complex behaviors often occur with therapeutic-range doses. Drug-drug interactions may be involved; for example, a pharmacokinetic drug-drug interaction that inhibits cytochrome P450 metabolism could potentiate zolpidem. A pharmacodynamic drug-drug interaction could also occur for example, taking a drug which depresses the central nervous system (CNS) or enhances GABAergic activity could produce additive effects [32]. While several plausible, if incomplete, explanations have been set forth to explain complex behaviors, no single explanation or agent can elucidate all of the cases. In a study of traffic stops and arrests for sleepdriving or other forms of impaired driving while taking the so-called “z-hypnotics,” including but not limited to zolpidem, it was found that impaired drives exhibited one or more of the following traits: they had higher-than-therapeutic serum concentrations of the drug while driving, they had failed to take the drug according to manufacturer's directions, or they had combined the sleeping agent with a CNS depressant or alcohol or both [33]. It should be noted that those taking z-drugs and detained for impaired driving are often unable to stand unassisted, and while they can speak, they have extremely poor motor control, in contrast to sleep walkers who can ambulate and maintain their balance.

In the case of sleepwalking, it has been suggested that such behavior is more common than generally believed, occurring in 1% to 15% of the general population and associated with sleep deficit—a common condition of insomniacs [34]. In a review of specific cases of sleep-related complex behaviors associated with zolpidem, these actions appear to be dose-dependent [30]. It may be that lower doses of zolpidem are associated with different rates of complex behaviors. For example, the literature reports a case of a patient who suffered from sleep eating with an extended-release formulation of zolpidem that resolved when changed to an immediate-release formulation [35]. Further studies are needed.

Recently the FDA has issued a change in product label for all zolpidem marketed products [36]. Reports have indicated that patient blood concentration levels may still be too high the morning after dose administration, resulting in residual somnolence and impairing activities that require alertness. These morning after effects have been shown to increase risk of car accidents and falls [37, 38]. Driving simulation and laboratory studies indicate that zolpidem blood concentration levels above 50 ng/mL are capable of impairing driving. Pharmacokinetic trials of zolpidem products reported a significant number of patients above this threshold the morning after administration. By lowering the recommended dose for these products, potential risk for these morning after effects may be reduced. It should be noted that Intermezzo's product label was not required to be changed due to it already being a lower dose formulation.

## 9. Abuse Potential

Zolpidem is a Schedule IV controlled substance in the United States according to the Controlled Substances Act. A retrospective study found that zolpidem had the potential for abuse and dependence in certain patients, particularly those with a history of substance abuse and those actively consuming multiple types of drugs [39], but it should be noted that low-dose, fast-acting zolpidem is a new product and was not included in this study. It is not clear if a low-dose, short-acting product would have less appeal to a potential abuser than higher-dose formulations. Zolpidem dependence with withdrawal symptoms has been reported in the literature, which was effectively managed with pregabalin [40].

Mechanistic animal studies on physical dependence and abuse liability of GABAA-*α*1-preferring drugs, such as zolpidem, have been equivocal. Some primate studies suggest that the GABAA-*α*1-subunit is involved in physical dependence, suggesting that the hypnotic and abuse liability activity of zolpidem might not be separated [41]. Other studies on genetic mice models suggest that subunits other than *α*1 (such as *α*3) are involved in reward-enhancing activity of various GABA agonists, including zolpidem [42].

Zolpidem does not appear to induce tolerance in insomniac patients, even with long-term use [43, 44]. The use of sublingual zolpidem 10 mg at bedtime over the course of one year did not induce tolerance in primary insomniacs [45], but it is uncertain whether a fast-acting sublingual formulation would exhibit a different tolerance profile.

## 10. Cancer

A retrospective health insurance database study (*n* = 14,950) in Taiwan found that zolpidem users had a greater risk than nonusers for developing any of several types of cancer (overall hazard ratio 1.68, 95% confidence interval, 1.57 to 3.56), with men at higher risk than women [46]. This risk increased with dose with greater use of zolpidem (defined here as ≥300 mg/year). Since this is a single retrospective study in a highly homogeneous population, it requires confirmation, particularly in light of such unusual findings. This study did not gather data from patients on smoking, alcohol consumption, or obesity, all of which may have contributed to higher cancer rates.

## 11. Zolpidem Use in Special Populations

### 11.1. Geriatric Patients

Zolpidem pharmacokinetics in general differs in younger and older patients; the elderly have an increased maximum serum concentration (*C*_max⁡_) and lower oral clearance rates, meaning that lower doses are recommended in individuals over the age of 60 [47]. A population-based cohort study found that ambulatory seniors were often prescribed supratherapeutic doses of zolpidem [48]. The recommended dose of zolpidem for geriatric patients is 5 mg/day (for long-acting formulations) and 1.75 mg/day for men and women of the fast-acting sublingual formulation [19].

### 11.2. Women

Insomnia is more common in women and may affect women differently than men [49]. Attempts have been made to correlate higher insomnia rates in women with greater rates of depression, mental health disorders, and hormonal disruptions, but, even taking those into account, women are more likely to experience insomnia than men [50]. Insomnia has been more closely correlated to pain and somatic symptoms in women than men [51]. Furthermore, insomnia prevalence in women increases with advancing age [50]. The apparent female predisposition to insomnia causes more elderly women than elderly men to take hypnotics and sedatives to facilitate sleep [52]. A Danish registry study (*n* = 10,000) found that female gender and advanced age were risk factors for the long-term use of zolpidem and other z-hypnotics [53]. Gender-specific dosing should be followed with zolpidem as drug clearance rates are slower in women than men. Women have a significantly higher serum concentration of zolpidem than men, as much as 40% greater, a factor that should be considered when making prescribing decisions [54]. In January of 2013, the FDA recommended that dosages be reduced for women of certain immediate-release zolpidem products (Ambien, Edluar, and Zolpimist) from 10 mg to 5 mg and extended-release zolpidem (Ambien CR) be dropped from 12.5 mg to 6.25 mg. The dosage of the lower-dose middle-of-the-night product (Intermezzo) remains unchanged as it was released to market with a lower dosage for women than men.

In a systematic review of the literature of driving studies involving a variety of sleeping agents (*n* = 14 studies), differences in driving ability by gender were noted the morning after taking a sleeping agent for flurazepam 30 mg and zolpidem 10 mg administered in the middle of the night, but not for ramelteon 8 mg, lormetazepam 1 and 2 mg, zaleplon 10 and 20 mg, and zopiclone 7.5 mg [55]. Women seem to be affected more by these drugs in terms of driving than men. It must be noted that these authors compared a variety of studies using different drugs, different study methodologies, and different patient populations; for instance, some studies enrolled only men or only women.

Zolpidem is classified as a category C drug for use during pregnancy, meaning that the risk of its use in this population cannot be ruled out. Zolpidem has been associated with adverse pregnancy outcomes in a population-based study in Taiwan [56].

## 12. Clinical Perspective

Since many sleeping products are available on the market, including several zolpidem formulations, the arrival of a new product may seem unnecessary. Fast-acting, low-dose sublingual zolpidem is important because it is indicated specifically for middle-of-the-night wakefulness, a need not previously addressed by the current products. Middle-of-the-night wakefulness is a core symptom of insomnia and not a rare one; it is one of the most common ways that insomnia presents. Prior zolpidem formulations were long-acting products that required patients to have at least eight hours reserved for sleep upon taking the medication. Patients taking a long-acting formulation and then arising while the drug was still in effect are thought to be at greater risk for unexpected and complex behaviors, such as sleepdriving [19].

Though there are treatment options being researched for the morning after effects such as sublingual Flumazenil, a GABA_A_ receptor antagonist, these may not be suitable for all patients and additional medications may not be feasible [57]. Therefore, a fast-acting, low-dose product that is effective only for a four-hour time period meets a need not served by any of the other formulations.

When treating insomnia, clinicians should first rule out any underlying conditions that might be causing sleeplessness. Many patients with mild or occasional insomnia may benefit from nonpharmacological treatment options, such as lifestyle modifications or changes in sleep hygiene. There remain many patients who will require pharmacological therapy to manage the distressing symptoms of primary insomnia. Sleep aids are powerful agents and should be prescribed with patient education so that patients understand their potential risks as well as benefits. Considerable direct-to-consumer advertising of sleeping pills may encourage in some consumers a false notion that sleep aids are harmless drugs that can be consumed casually. Thus, clinicians must instill in patients the important concept that drugs to fight insomnia are powerful agents to be taken only as directed, when needed, and under medical supervision.

Since this is a new indication for zolpidem, clinicians should counsel patients about this agent and how to take it. In particular, a gender-specific (and age-specific) dosing regimen should be emphasized. The gender-specific dosing for fast-acting low-dose zolpidem also appears to recognize a previously overlooked consideration, namely, that women metabolize zolpidem differently than men. Since more women than men suffer from insomnia and more women take sleep aids, this is not a trivial consideration.

As with all drugs in this class and use in this application, adequate caution is required regarding possibly allergy to the drug substance, adverse effects, drug-drug interactions, and other potential problems that should be adequately reviewed before prescribing or taking.

## 13. Conclusion

Fast-acting sublingual zolpidem tartrate (Intermezzo) is a new low-dose formulation designed to specifically address the somnipathy of middle-of-the-night wakefulness. It is the first FDA-approved drug exclusively for this use. Since middle-of-the-night wakefulness is a frequent core symptom of primary insomnia, this new agent addresses an important need. The fast-acting low-dose sublingual formulation offers an interesting new option for the right patients.

## Figures and Tables

**Table 1:** Zolpidem products currently marketed in the United States for treatment of insomnia. Zolpidem 10 mg tablets are marketed in Australia under the trade name Stilnox.

Brand Name	Manufacturer	Formulation	Indication	Strength(s)
Ambien [58]*	Sanofi	Immediate-release tablet	Difficulty with sleep initiation	5 and 10 mg

Ambien CR [59]	Sanofi	Controlled-release tablet (sometimes called extended-release or modified-release Ambien)	Difficulty with sleep onset and/or sleep maintenance	6.25 and 12.5 mg

Edluar [60]	Meda	Sublingual tablet	Difficulty falling asleep	5 and 10 mg

Intermezzo [19]	Transcept/Purdue Pharma	Fast-acting sublingual tablet	Middle-of-the-night wakefulness and difficulty returning to sleep	1.75 and 3.5 mg

Zolpimist [61]	NovaDel/ECR	Oral spray	Difficulty with sleep initiation (also being studied for middle-of-the-night wakefulness)	5 mg

All brand names are trademarks or registered trademarks of the manufacturer or drug owner.

*The patent on the Ambien immediate-release formulation has expired and generic products have been introduced into the market.

**Table 2:** Pharmacokinetic parameters for zolpidem formulations.

Brand name	AUC(ng·h/mL)	*C*_max⁡_ (ng/mL)	*T*_max⁡_ (hours)	*T*_1/2_ (hours)	Protein binding
Nonbenzodiazapines					
Ambien* 5 and 10 mg, respectively [58]		59 (range 29–113) and 121 (range 58–272)	1.6 for both	2.6 (range 1.4–4.5) and 2.5 (range 1.4–3.8)	92.5 ± 0.1%

Ambien CR 12.5 mg [59]	740 ng·hr/mL (range: 295 to 1359 ng·hr/mL)	134 (range 68.9–197)	1.5	Similar to above	92.5% ± 0.1%

Edluar 10 mg [60]		106 (range 52–205)	1.4	2.65 (range 1.75–3.77)	92.5% ± 0.1%

Intermezzo 3.5 mg [21]	Healthy Men: 198 Women: 296 *Elderly & hepatic impaired may vary	3.5 mg: men 53 and women 77. *Elderly & hepatic impaired may vary	0.6 to 1.25	2.5 (range 1.4 to 3.6)	93% ± 0.1%

Zolpimist 5 and 10 mg [61]		114 (range 19–197) and 210 (range 77–401), respectively	0.9 for both	2.7 (range 1.7–5.0) and 3.0 (range 1.7–8.4), respectively	92.5% ± 0.1%

Benzodiazapines					
Triazolam (Halcion) [62]		1–6	2	1.5–5.5	

Temazepam (Restoril) (30 mg) [63]		666 to 982 ng/mL (mean 865 ng/mL)	1.2 to 1.6 hours (mean 1.5 hours)	4–18	96

All brand names are trademarks or registered trademarks of the manufacturer or drug owner.

*The patent on the Ambien immediate-release formulation has expired and generic products have been introduced into the market.
